# Phytoplankton Under Pressure: Temperature, Precipitation and Cyanobacterial Blooms as Drivers of Chytrid Infections

**DOI:** 10.1111/1758-2229.70224

**Published:** 2025-11-07

**Authors:** Martyna Budziak, Doris Ilicic, Hans‐Peter Grossart, Wojciech Krztoń, Edward Walusiak, Janusz Fyda, Elżbieta Wilk‐Woźniak

**Affiliations:** ^1^ Institute of Nature Conservation Polish Academy of Sciences Kraków Poland; ^2^ Department of Plankton and Microbial Ecology Leibniz Institute of Freshwater Ecology and Inland Fisheries Stechlin Germany; ^3^ Institute of Biochemistry and Biology University of Potsdam Potsdam Germany; ^4^ Institute of Environmental Sciences Jagiellonian University in Kraków Kraków Poland

**Keywords:** abiotic parameters, chytrid occurrence, green‐algae, host–parasite, infection prevalence, parasitic fungi

## Abstract

The area of fungal parasitism is attracting growing attention because of its great importance for aquatic organisms and their community dynamics. Despite increasing interest in this area, few studies have addressed baseline data on occurrence and environmental factors associated with chytrid parasite infections in natural ecosystems. This work provides insights into occurrence, prevalence, and dynamics of parasitic infections by studying three freshwater reservoirs over a period of 6 years. Chytrid infections were detected in each of the studied water bodies, infecting species of cyanobacteria, green algae and diatoms. However, recurring and prevalent infections were observed in only one water body, which is classified as a natural aquatic ecosystem. The recorded infection prevalence (IPC) ranged between 0% and 20%, while the mean infection severity remained low. Infection rates were highest in summer and most prominent during cyanobacterial blooms. Yet, the most infected group of phytoplankton consisted of green algae. GLM revealed a significantly positive correlation between IPC and water temperature and precipitation. Overall, these results demonstrate the dynamic nature of chytrid infections, which are shaped by multiple environmental factors across space and time.

## Graphical abstract

Chytrid parasites of phytoplankton infect a wide range of species, including green algae, diatoms, and cyanobacteria. Infection patterns varied, indicating that chytrid occurrence is highly dynamic in space and time. Warmer temperatures, increased rainfall and cyanobacterial bloom events coincided with enhanced infection prevalence on green algae, suggesting that parasite–host interactions may be sensitive to climate‐driven changes in freshwater conditions.

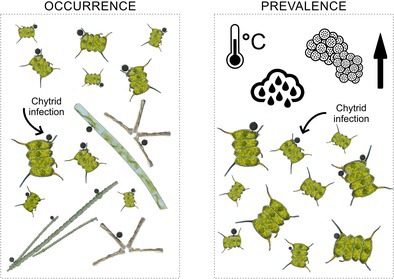

## 1 Introduction

The field of aquatic parasitism receives increasing attention from aquatic scientists, as parasites play an integral role in the food web (Kagami, de Bruin, et al. 2007; Rasconi et al. 2012; Frenken et al. 2017; Grossart et al. 2019; Thongthaisong et al. 2025). This has been partially driven by the increasing recognition and investigation of previously undocumented fungal diversity, commonly referred to as ‘dark matter’ fungi (Grossart et al. 2016), owing to their significant role in aquatic food web dynamics. Zoosporic parasites represent another ecologically significant group, characterised by a motile, free‐living stage during which flagellated zoospores actively navigate through the water column, searching for suitable hosts (Ibelings et al. 2004; Sime‐Ngando 2012). Among fungal taxa, members of the order Chytridiales (commonly known as chytrids) overlap with both of these groups, frequently exhibiting parasitic lifestyles by infecting phytoplankton. Most available data on these fungi have been documented in temperate regions (Shearer et al. 2007). However, our current understanding of chytrid parasites, their interactions with phytoplankton, and environmental drivers remains limited and requires further investigation.

Both biotic and abiotic environmental factors are known to influence chytrid parasites (Wolinska and King 2009). In temperate water bodies, abiotic drivers of parasitic infection, such as temperature and precipitation, are primarily associated with changing seasons. However, accelerating global warming is predicted to alter these effects, leading to additional shifts in climate dynamics (Lee et al. 2023). A previous study has hypothesised that climate change may lead to either an increase or decrease in parasite pressure, depending on the context‐specific interactions between hosts, parasites, and their environment (Marcogliese 2025). Notably, temperature has been identified as a key abiotic factor capable of shaping parasite‐free refuges for hosts (Catlett et al. 2023; Gsell et al. 2023), further highlighting its regulatory role. Yet with the increase in temperature, both the transmission rates and the virulence of pathogens and parasites are expected to rise (Marcogliese 2008; Lafferty and Mordecai 2016). This trend may also apply to chytrid parasites, as both experimental and environmental studies have documented a positive relationship between rising temperatures and increased infection prevalence and chytrid richness (Agha et al. 2018; Gsell et al. 2022; Rajarajan et al. 2025). Global warming also impacts precipitation patterns, intensifying severe rainfall events and/or drought periods (Lee et al. 2023). Intense rainfall may increase nutrient flushing into water bodies, accelerating eutrophication of aquatic ecosystems (Fong et al. 2020). As a consequence, the composition of phytoplankton communities can change, leading to alterations of parasite hosts (Frenken et al. 2021). This, in turn, can trigger a higher prevalence of chytrid parasite infections (McKenzie and Townsend 2007), which may act as a factor in the process of phytoplankton community assembly and, thus, influence biodiversity.

Several studies have suggested that chytrids exhibit a higher preference for bigger host cells and colonial species (Ibelings et al. 2004; Kagami, de Bruin, et al. 2007; Sime‐Ngando 2012), and can target specific host cells, for example, akinetes during cyanobacterial blooms (Gerphagnon et al. 2013). Additionally, parasite densities are more prominent while their host populations are highly abundant, suggesting a positive correlation between infection prevalence and algal blooms, as the availability of suitable hosts is crucial for parasite presence, development, transmission and infectivity (Ibelings et al. 2011; Rasconi et al. 2012; Sime‐Ngando 2012). Beyond host size and density, some studies also suggest that the physiological condition of the host plays an important role in the infection success. Weakened algae experiencing environmental stress, such as elevated temperature and/or high light intensity, tend to exhibit increased vulnerability to infection (Frenken et al. 2016; Wierenga et al. 2022). Yet, infection success is not always linked to host stress, as certain studies indicate that parasites may actually thrive better in healthy hosts (Van den Wyngaert et al. 2014). High rates of chytrid infections can also cause host species decline, leading to a shift in phytoplankton community composition from large, inedible filamentous and colonial forms to smaller, easy‐to‐handle forms (Agha et al. 2016; Frenken et al. 2017, 2020; Abonyi et al. 2024).

In the face of global warming and advanced eutrophication, harmful cyanobacterial blooms are a pivotal topic, as they are predicted to proliferate in warming aquatic habitats (Paerl et al. 2011; Wilk‐Woźniak et al. 2024). These blooms can alter energy transfer and community dynamics (Krztoń et al. 2019, 2025), as cyanobacteria often serve as unfavourable or even inedible prey for zooplankton (Frenken et al. 2018; Thongthaisong et al. 2022, 2025). However, an alternative pathway known as ‘mycoloop’ has emerged, linking phytoplankton to zooplankton through fungal parasites (Kagami et al. 2014; Frenken et al. 2018). These parasites produce zoospores that are released into the environment and can serve as a nutritious food source for zooplankton, altering food web structure and, ultimately, ecosystem dynamics (Gsell et al. 2022). Therefore, we want to know whether cyanobacterial blooms can be effectively controlled by chytrid parasites in nature.

Here, we analysed data collected during 6 years of environmental monitoring of three shallow water bodies located in Southern Poland. The aim of the study was to infer occurrence, prevalence and dynamics of parasitic infections on phytoplankton under natural conditions in these eutrophic water bodies. Specific goals included: (i) studying temporal distribution of parasites; (ii) determining host species and the percentage of infected phytoplankton populations; (iii) assessing potential relations between parasite features and environmental factors such as nutrient concentration, host biomass, bloom presence and meteorological factors. We hypothesised that (i) the occurrence of chytrid infections and high infection prevalence would correspond to high phytoplankton biomass, particularly during cyanobacterial blooms; and that (ii) infection prevalence would increase with increasing temperature and nutrient concentrations.

## 2 Materials and Methods

### 2.1 Study Site

The study was conducted in three water bodies: an oxbow lake of the Vistula River (Tyniec; T) and two artificial ponds: Podkamycze 1 (P1) and Podkamycze 2 (P2) located in the area of Kraków (Southern Poland). All three water bodies are shallow and eutrophic, with cyanobacterial blooms occurring annually (Table 1). They are located in close proximity to each other, ensuring similar weather conditions that are unlikely to influence differences in their functioning.

**TABLE 1:** Basic information about the studied water bodies.

	Tyniec	Podkamycze 1	Podkamycze 2
Geographical coordinates	50°01′28.1″ N, 19°48′47.7″ E	50°05′11″ N, 19°50′01.6″ E	50°04′59.6″N, 19°50′05.4″ E
Type of water body	Natural	Artificial	Artificial
Max depth (m)	2.75	3.0	2.5
Area (ha)	8.61	16.82	17.28

### 2.2 Sampling

Samples were collected monthly or fortnightly from April to October each year (69 time points) between 2019 and 2024 (6 years, altogether 205 samples). Sampling has been performed with a 5 L Bernatowicz sampler at a central point of each water body at a depth of 1 m. Phytoplankton samples for microscopic analyses were concentrated over a 10 μm plankton mesh from an initial volume of 10 L, and the remaining ~60 mL of concentrates were fixed with Lugol's iodine solution. Water temperature and conductivity were measured in situ using a YSI 6600 V2 multiparameter probe (YSI, USA). Water samples for nutrient and pH analysis were immediately transported to the laboratory of the Institute of Nature Conservation Polish Academy of Sciences for further analysis. The dataset of physicochemical water parameters is provided in Table S1.

### 2.3 Environmental Factors

Meteorological factors, including precipitation (PP), cloud cover (CC) and mean wind speed (WS), were obtained from the Institute of Meteorology and Water Management National Research Institute in Poland open‐access database (Meteomodel.pl 2024) https://meteomodel.pl/.

Analyses of ion concentrations (nitrate—NO_3_^−^, phosphate—PO_4_^3−^, ammonium—NH_4_^+^) were performed using a Dionex IC25 ICS‐1000 ion chromatograph (Dionex, USA). The pH was measured using a Greisinger G1500 pH meter with a GE 114 probe (Greisinger, Germany).

### 2.4 Phytoplankton and Chytrids Analyses

Qualitative and quantitative analyses of phytoplankton were conducted using a Zeiss Jenaval light microscope at magnifications ranging from 40× to 400× in a 0.5 mL chamber. Phytoplankton identification was carried out based on taxonomic keys listed in Wilk‐Woźniak (2009) and supplemented by Komárek (2013). Biomass was estimated as biovolume by comparing specimens to their corresponding geometric shapes, following the method outlined by Rott (1981).

Phytoplankton Lugol‐fixed samples were used to determine chytrid presence by double‐staining two 1 mL subsamples, which were previously de‐stained using sodium thiosulfate. To visualise chytrid sporangia, samples were stained with Calcofluor White (CFW) and Wheat Germ Agglutinin, conjugated to Alexa Fluor 488 (WGA) fluorescent dyes according to the protocol described by Rasconi et al. (2009) and Klawonn et al. (2023). Each sample was analysed in duplicate under an inverted microscope (Nikon eclipse Ti2 and Olympus IX71, fluorescence channels CFW: 387/11 nm excitation and 442/46 nm emission, WGA: 482/35 nm excitation and 536/40 nm emission), according to the Utermöhl method (Utermöhl 1958). The analysis involved sample pre‐screening for the presence of chytrid fungi. If pre‐screening detected infections, 300 cells of infected phytoplankton taxa were counted to determine the proportion of infected cells. Active infections were identified by the presence of mature sporangia, while empty sporangia indicated the end of an infection. The exact number of hosts with mature or empty sporangia was counted and photographed. The subsequent steps of the analysis included calculating infection prevalence (IPC) according to the formula: IPC (%) = [(𝑁𝑖/𝑁𝑡) × 100], where 𝑁𝑖 is the number of cells infected by chytrids and 𝑁𝑡 is the total number of cells. Additionally, we calculated infection severity, which translates to: IS = 𝑁ps/𝑁i, where 𝑁ps is the number of attached sporangia, and 𝑁𝑖 is the number of infected individuals within a host population.

For further analysis, data were classified according to cyanobacterial bloom presence: the pre‐cyanobacterial bloom period (April–June) and the cyanobacterial bloom period (July–October), based on the onset of the bloom as defined by Huisman et al. (2018).

### 2.5 Statistical Analyses

To test whether physical parameters (water temperature, conductivity, and pH), chemical parameters (ion concentrations), and phytoplankton biomass differed between the studied water bodies, a Kruskal–Wallis test was employed for each variable separately. When significant differences were detected, a post hoc Dunn's test with Bonferroni correction was conducted to determine pairwise differences between the water bodies.

PCA analysis was performed to assess the contribution of the principal components, ensuring that the model adequately represented the data (Figure S1). Factors were checked for collinearity and included or excluded as needed, by comparing variance inflation factors (VIF). Finally, generalised linear models (GLMs) were fitted to assess factors influencing infection dynamics in the studied reservoirs. A GLM with a quasi‐Poisson distribution was used to examine infection prevalence, with temperature, precipitation, and cyanobacterial bloom presence as explanatory variables. Next, a GLM with a Gaussian distribution was used to analyse the effect of the above‐mentioned factors on host species biomass as the dependent variable, in order to account for their possible influence on the host. Data processing, statistical analyses and visualisations were performed in RStudio (R Core Team 2022). Significance levels were set at *p < 0.05*.

## 3 Results

**FIGURE 1:**
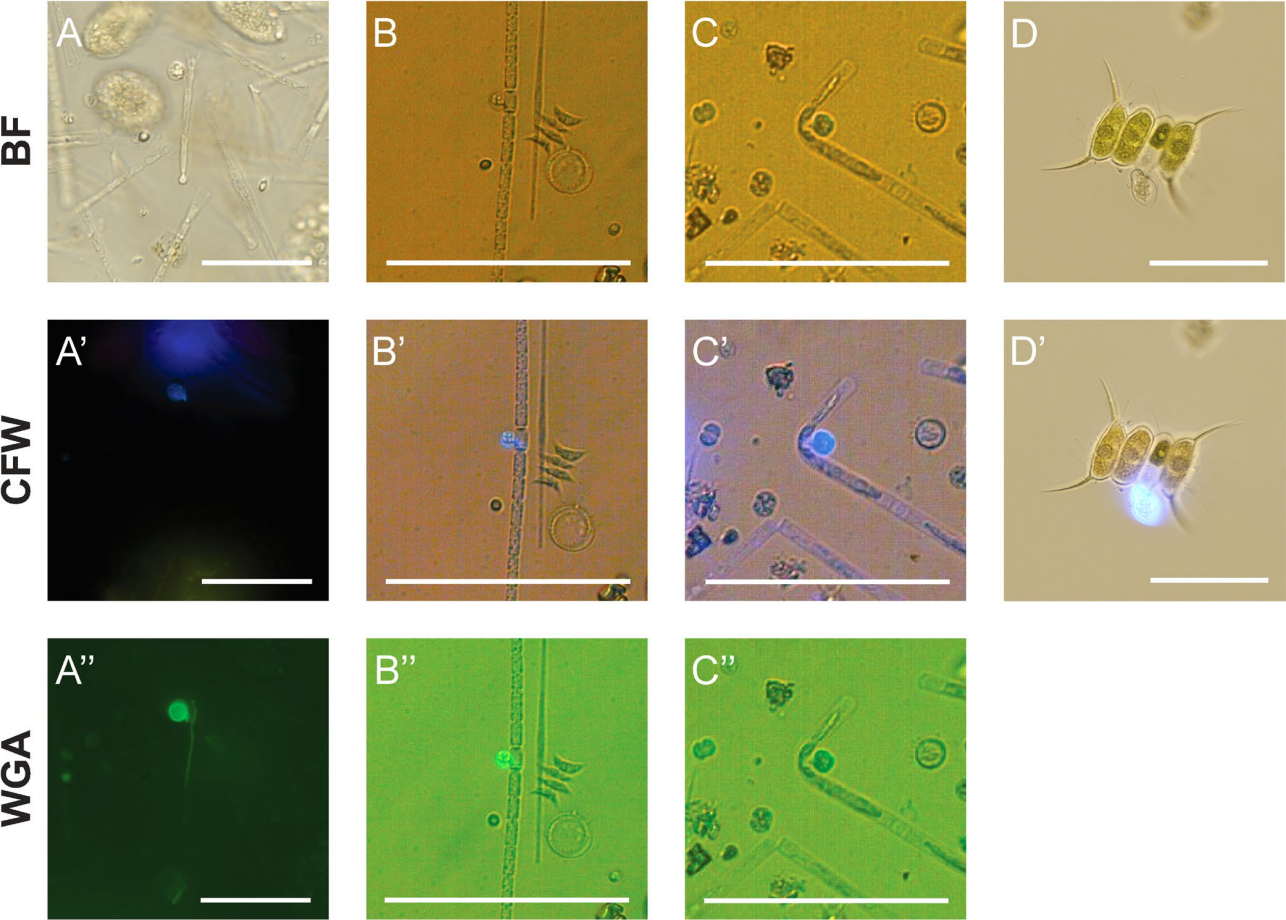
Examples of microscopic micrographs of phytoplankton hosts with attached chytrid parasites obtained during our seasonal surveys in (A) Podkamycze 1— *Asterionella formosa* , (B) Podkamycze 2—*Aphanizomenon* cf. *gracile*, (C) Podkamycze 2—*Mougeotia* sp. and (D) Tyniec—*Desmodesmus* cf. *armatus*. The scale bar represents 50 μm. Abbreviations: BF, bright field; CFW, Calcofluor White; WGA, Wheat Germ Agglutinin.

Chytrid infections were found in 40 samples across the three examined water bodies, with 37 from Tyniec oxbow lake, one sample from Podkamycze 1, and two from Podkamycze 2. Both mature and empty sporangia were observed. The infected phytoplankton hosts belonged to cyanobacteria, diatoms and green algae. These include: *Aphanizomenon gracile* Lemmermann (cyanobacteria), *Asterionella formosa* Hassall (diatoms), *Desmodesmus* spp. (green algae) and *Mougeotia* sp. (green algae—desmid). In Podkamycze 1, the only infected species was the diatom *A. formosa* (Figure 1A), with an IPC of 11.6%, observed in September 2021. In Podkamycze 2, the infected hosts included *Desmodesmus* spp. and *Mougeotia* sp. (chlorococcal and filamentous green algae, respectively), and *Aphanizomenon* spp. including *A. gracile* (cyanobacteria; Figure 1B,C). At the beginning of September 2024, the IPC in Podkamycze 2 reached 0.02% for *Desmodesmus* spp., 3.2% for *Aphanizomenon* spp., and 1.7% for *Mougeotia* sp. Two weeks later, infections persisted in *Desmodesmus* spp. and *Aphanizomenon* spp., with IPC values of 0.02% and 2.2%, respectively. In Tyniec oxbow lake, *Desmodesmus* spp. was the only infected host, exhibiting recurrent infections with an average infection rate of 4.71% ± 4.3% (mean ± SD; Figure 1D).

Weather conditions, including cloud cover, precipitation and wind speed, remained consistent across the studied water bodies due to their close proximity. The highest average precipitation and wind speed were recorded in 2024 (Table 2).

**TABLE 2:** Weekly average values of selected meteorological parameters representing the weather conditions across studied water bodies, April–October 2019–2024.

	Cloud cover (oktas)				Precipitation (mm)				Wind speed (m/s)			
	AV	SD	Min	Max	AV	SD	Min	Max	AV	SD	Min	Max
2019	4.5	1.26	2.39	6.59	2.37	2.90	0	10.3	2.91	0.77	1.7	4.56
2020	4.44	1.08	2.73	6.3	1.45	1.63	0.03	6.3	2.72	0.64	1.86	3.64
2021	4.36	1.29	2.33	5.93	4.60	4.60	0.21	12.3	2.93	1.09	0.76	4.1
2022	4.97	0.82	4.01	6.04	2.70	2.70	0.37	8.23	2.97	0.71	2.3	4.26
2023	4.74	1.35	2.7	6.59	2.19	2.78	0	9.57	2.25	0.68	1.3	3.47
2024	4.77	0.99	2.44	6.6	5.35	4.98	0	18.6	3.71	2.42	2.01	9.21

Abbreviations: AV, average; min–max, range of parameters; SD, standard deviation.

**FIGURE 2:**
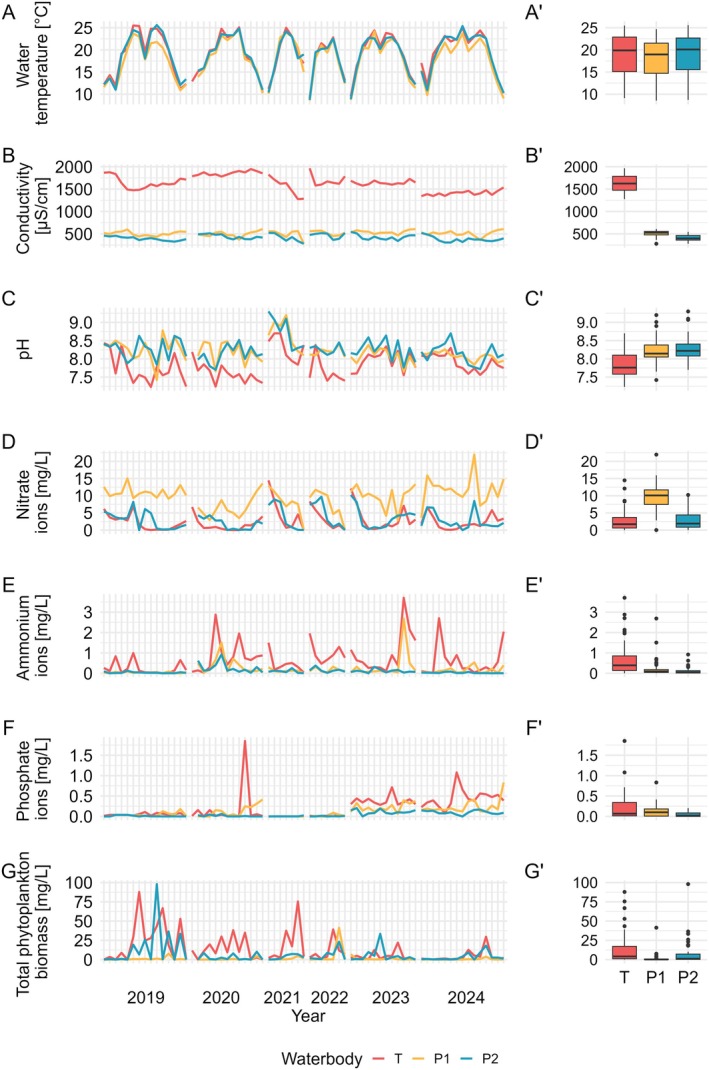
Seasonal trends in (A) water temperature (°C), (B) conductivity (μS/cm), (C) pH, (D) nitrate (mg/L), (E) ammonium (mg/L), (F) phosphate (mg/L), and (G) total phytoplankton biomass (mg/L) in Tyniec, Podkamycze 1 and Podkamycze 2, measured from April to October 2019–2024. Total values of explanatory variables for selected water bodies.

Water temperature exhibited similar patterns across the studied water bodies throughout the entire study period (Figure 2A), with no significant differences observed between them (Figure 2A). Similarly, conductivity levels followed a consistent pattern across all water bodies, although their absolute values varied (Figure 2B). The Tyniec oxbow lake consistently recorded the highest conductivity levels, followed by Podkamycze 2 and Podkamycze 1, with each water body being statistically different from the others (*p* < 0.001; Figure 2B). In contrast, pH levels fluctuated over the 6 years (Figure 2C), with Tyniec showing significantly lower pH values compared to Podkamycze 1 and 2 (*p* < 0.001; Figure 2C). In terms of ion concentrations, notable seasonal and inter‐annual fluctuations were detected over the duration of the study period (Figure 2D–F). Nitrate ion concentrations were observed to be the highest in Podkamycze 1 (*p* < 0.001), followed by Podkamycze 2 and Tyniec, with no significant differences between the latter two (Figure 2D). Similarly, ammonium ion concentrations were significantly higher in Tyniec (*p* < 0.001) compared to Podkamycze 1 and Podkamycze 2 (Figure 2E). In the case of phosphate ions, a significant difference was detected (*p* < 0.001), with Podkamycze 2 exhibiting the lowest concentrations. Post hoc analysis revealed no significant difference between Podkamycze 1 and Tyniec (Figure 2F).

**FIGURE 3:**
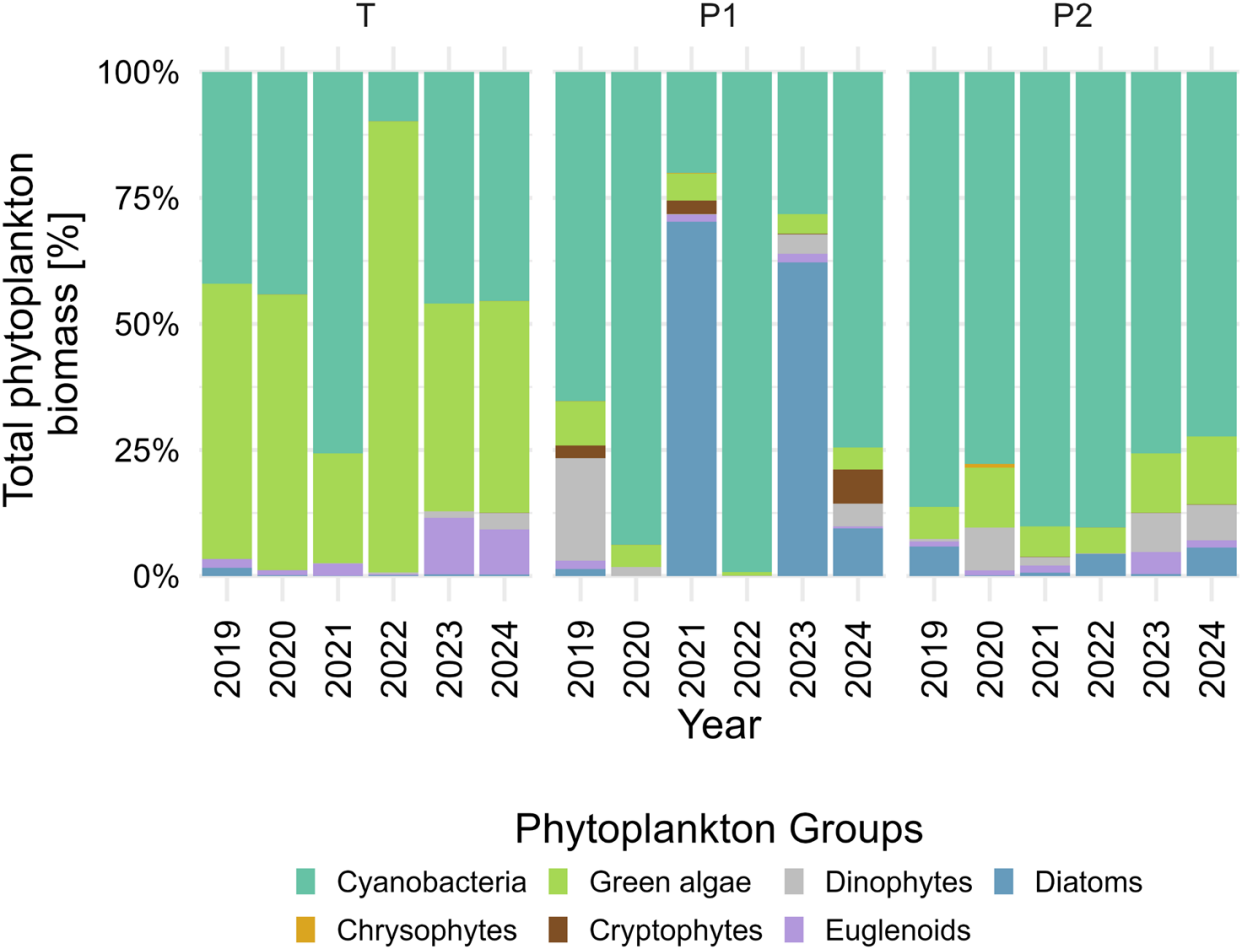
Relative proportion of biomass in phytoplankton groups in the studied water bodies (Tyniec, Podkamycze 1, Podkamycze 2) during the study period (2019–2024).

Phytoplankton biomass and composition differed between the studied water bodies (Figure 2G; Figure 3), with Tyniec having the highest total biomass compared to other water bodies (*p* < 0.001; Figure 2G). The total biomass in Tyniec oxbow lake reached 876.2 mg/L and the phytoplankton composition was dominated by green algae and cyanobacteria (representing 50% and 46% of the total biomass, respectively). Notably, *Desmodesmus* spp., the only observed host of chytrid parasites in Tyniec oxbow lake, constituted 49% of the total biomass and almost the entire biomass (99%) of the green algae community. In Podkamycze 1, the total biomass equalled 88.8 mg/L and the major phytoplankton phyla were cyanobacteria (76%) and diatoms (15%). During September 2021, when chytrid infection was recorded, the total phytoplankton biomass reached 4.694 mg/L, with *A. formosa*, the only observed infected species, accounting for 4.013 mg/L, or approximately 85% of the total phytoplankton biomass. In Podkamycze 2 the biomass accounted for 429.9 mg/L and cyanobacteria dominated the phytoplankton composition (83% of the total biomass), with other groups contributing less than 10%. At the beginning of September, when chytrid infections were detected, the total phytoplankton biomass was 18.138 mg/L. During this period, the biomass of infected taxa was very low, with *Desmodesmus* spp. contributing 0.007 mg/L, *Mougeotia* sp. 0.107 mg/L, and *Aphanizomenon* spp. 0.063 mg/L. Two weeks later, *Desmodesmus* spp. and *Aphanizomenon* spp. persisted as hosts, with biomasses of 0.024 and 0.007 mg/L, respectively, while *Mougeotia* sp. was no longer recorded.

The dominant cyanobacterial community in Tyniec oxbow lake consisted primarily of *Microcystis aeruginosa* (Kützing) Kützing. In Podkamycze 1, *Aphanizomenon flos‐aquae* Ralfs ex Bornet & Flahault and *Aphanizomenon gracile* were predominant, while in Podkamycze 2, the community was composed of *Aphanizomenon gracile, Microcystis aeruginosa*, and *Microcystis wesenbergii* (Komárek) Komárek ex Komárek. Phytoplankton blooms occurred annually from July to October in all reservoirs.

Due to non‐recurring infection observations in Podkamycze 1 and 2, these water bodies were excluded from statistical analyses, which were limited to Tyniec oxbow lake, where infections were present in each year of sampling.

**FIGURE 4:**
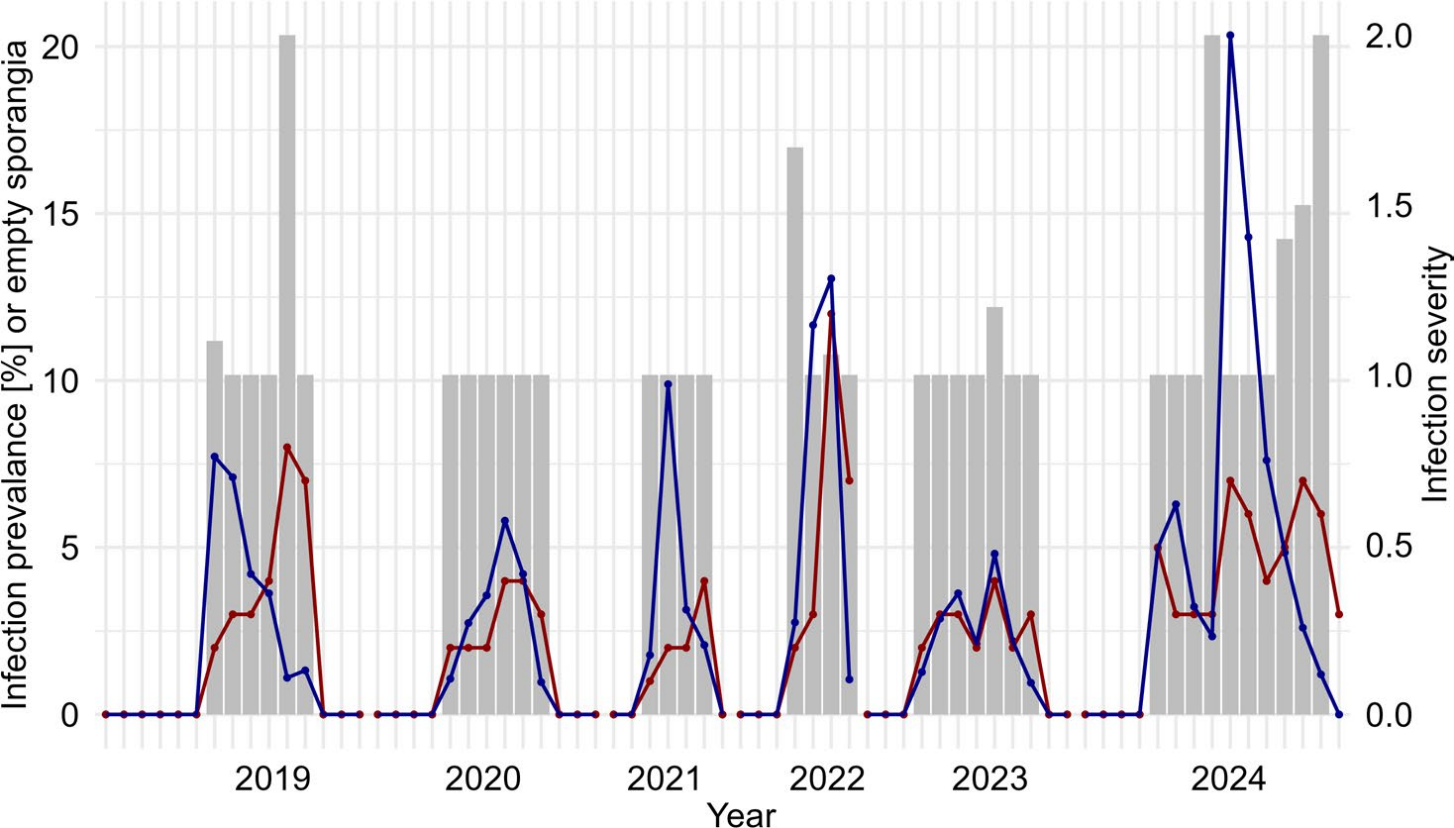
Time series of infection prevalence (IPC %, blue, left), empty sporangia (red, left), and infection severity (grey, right) in Tyniec oxbow lake, over the study period from April to October 2019–2024.

The minimum recorded IPC in Tyniec oxbow lake, if present, was 1%, while the maximum IPC reached 20% (mean ± SD = 4.71% ± 4.3%). Infections exhibited similar dynamics throughout the years 2019–2024, starting in early summer (June–July) and ending in autumn (September–October). The infections typically followed a consistent pattern: beginning with around 1%–3% infected cells, peaking at approximately 8%, and then declining, as the number of empty sporangia increased (Figure 4). Infection severity was generally low, ranging from 1 to 2 sporangia per coenobium of *Desmodesmus* (mean ± SD = 1.14 ± 0.3 sporangia per host; Figure 4).

There were no statistically significant differences in IPC between years in Tyniec oxbow lake; however, the presence of cyanobacterial blooms was associated with significantly higher IPC. Moreover, a significantly positive relationship was found between IPC and water temperature as well as precipitation (Table 3). In contrast, no significant differences were found between IPC and conductivity, pH, nitrate ions, ammonium ions, phosphate ions, wind speed, cloud cover or the biomass of the host. Similarly, the GLM analysis indicated that none of the tested environmental variables had a significant effect on host biomass (Table 3).

**TABLE 3:** Results of the generalised linear model analysis of infection prevalence (IPC) and *Desmodesmus* spp. biomass as dependent variables with water temperature (°C), precipitation (mm), pH, nitrates (mg/L), phosphates (mg/L) and cyanobacterial bloom presence as explanatory variables on observation data in Tyniec oxbow lake.

	Estimate	SE	*t*‐value	Pr(> |*t*|)
*Infection prevalence*				
Intercept***	−3.67	1.04	−3.52	< 0.001***
Precipitation*	0.06	0.03	2.29	< 0.05*
Water temperature**	0.15	0.05	−3.24	< 0.01**
Cyanobacterial bloom presence**	1.45	0.53	2.72	< 0.01**
*Host biomass*				
Intercept	48.543	32.25	1.505	0.137
Precipitation	−0.007	0.394	−0.018	0.986
Water temperature	0.293	0.353	0.829	0.410
pH	−5.981	4.002	−1.495	0.140
Nitrates	−0.191	0.060	−0.315	0.754
Phosphates	−6.176	4.562	−1.354	0.181
Cyanobacterial bloom presence	1.509	3.345	0.451	0.653

*Note:* Bold values indicate significance levels: *p < 0.05, **p < 0.01, ***p < 0.001.

## 4 Discussion

All three studied water bodies belong to the most common type of stagnant water bodies on Earth, which are less than 3 m deep and smaller than 0.1 km^2^ (Reynolds 2004, 2006). Our assessment of six years of environmental changes in these eutrophic water bodies revealed that chytrid parasite infections on phytoplankton were apparent and repetitive in only one of them (Tyniec oxbow lake), while the other two (Podkamycze 1 and Podkamycze 2) exhibited only unprecedented occurrences. The exhaustive and detailed examination of the environmental conditions present in each of these reservoirs revealed differences in conductivity, pH and nutrient levels followed by differences in phytoplankton biomass and community composition with some seasonal fluctuations throughout the entire study period.

We observed infections on the cyanobacterium *A. gracile* (Podkamycze 2), the diatom *A. formosa* (Podkamycze 1), and the green algae *Desmodesmus* spp. (Tyniec, Podkamycze 2) and *Mougeotia* sp. (Podkamycze 2). Although chytrids are known to commonly parasitise cyanobacteria and diatoms, including *A. formosa* (Van Wichelen et al. 2010; Rasconi et al. 2012; Rohrlack et al. 2015; Gsell et al. 2022; Danz and Quandt 2023), we observed infections on diatoms and cyanobacteria only occasionally. In addition, we recorded chytrids infecting green algae, that is, *Mougeotia* sp. and *Desmodesmus* spp., which have previously been reported as hosts for chytrid parasites (Seto et al. 2023). Notably, chytrid infections of *A. gracile* were observed for the first time (compare to Van den Wyngaert et al. 2025). The majority of infections were found on the green algae *Desmodesmus* spp., with nearly 55% of samples from Tyniec oxbow lake showing signs of infection. Chytrid infections on *Desmodesmus* spp. occurred primarily in summer and autumn, suggesting that only from early summer onward host biomass and community composition became suitable to sustain detectable chytrid populations. However, no apparent relationship between the host biomass and IPC was found. This is contrary to the first hypothesis of this study. Infection prevalence and severity observed in this study were generally lower or comparable to those reported in similar field surveys (Sime‐Ngando 2012; Gsell et al. 2022; Van den Wyngaert et al. 2022; Ilicic et al. 2024), where IPC varied from 0% to 90%, and individual host cells could carry up to four sporangia.

Chytrid fungi can occur across a wide range of osmotic conditions (Sime‐Ngando 2012; Ilicic et al. 2024), however, their presence in our study was most pronounced in the Tyniec oxbow lake, an aquatic system with relatively high conductivity levels (1.63 ± 0.15 mS/cm) compared to other sites (Gsell et al. 2022; Van den Wyngaert et al. 2022). Moreover, pH and nutrient availability also played an important role in shaping chytrid distribution. In particular, chytrids were most abundant at pH 7.8 in Tyniec, compared to the more alkaline conditions (8.2–8.3) in Podkamycze 1 and 2. These values fall within previously reported chytrid tolerance ranges (Van den Wyngaert et al. 2022), yet experimental studies have shown that growth declines at more extreme pH values, suggesting a potential advantage near neutral pH (Gleason et al. 2011). Across the study sites, nutrient concentrations differed, while chytrid occurrence also varied over time and space. Tyniec oxbow lake exhibited relatively high phosphate and ammonium concentrations, coinciding with higher parasite occurrence contrasting both Podkamycze 1 and 2. Environmental parameters and chytrid occurrences varied spatiotemporally, and detections were limited. Consequently, no robust statistical models could be applied, and the patterns reported here represent associations rather than causal relationships.

Organisms experience both seasonal fluctuations in their abiotic environment (Gsell et al. 2022; Van den Wyngaert et al. 2022) and broader ones driven by global environmental change (Altizer et al. 2013). The tolerance to a specific environmental factor is outlined by an optimum range, beyond which no response in a trait is observed (Gsell et al. 2023). In our study, occurrence and IPC were positively correlated with increasing temperature. However, IPC was not significantly related to nutrients, which only partially is in accordance with our second hypothesis. Chytrids were present in samples in which the temperature averaged 21.7°C ± 3.1°C. The highest noted temperature with the parasitic infection present was 25.4°C, while the lowest temperature was 13.1°C; however, at that time point the infection was in decline. These results possibly suggest the presence of fungal species better adapted to warmer waters. The positive relationship of temperature and infection prevalence was also observed in a laboratory study employing the bloom‐forming cyanobacterium *Planktothrix* spp. (Agha et al. 2018). Furthermore, similar trends have also been reported by Wierenga et al. (2022) in their work on *Planktothrix rubescens* parasitised by chytrid belonging to the order Rhizophydiales, where increased temperature leads to a greater increase in the prevalence of infection, and this effect was further enhanced by light levels.

Global warming and climate change are expected to alter precipitation and drought trends, both spatially and temporally increasing the odds of extreme weather events (Lee et al. 2023). Increased rainfall can promote the runoff of nutrients from the catchment area to water bodies increasing their nutrient concentrations and promoting eutrophication (Paerl and Huisman 2008). In our study, IPC was positively correlated with precipitation; however, discussing this parameter is challenging, as very few studies have addressed it (Maier and Peterson 2017). Our data showed a trend different from the one observed in another study on chytrid parasitism in diatom populations in the lower Columbia River, where a high IPC was associated with high host density, low precipitation and low orthophosphate concentrations (Maier and Peterson 2017). Precipitation in general can either directly or indirectly influence water quality, habitat conditions, species composition, and overall ecosystem health. These impacts are often interconnected and can vary depending on the scale, intensity, and frequency of rainfall events (Akhtar et al. 2021). Therefore, it is difficult to conclusively determine the role of precipitation in our dataset, and further research is needed to better understand its influence on chytrid parasitism and ecosystem dynamics.

Several studies highlight the importance of host abundance in chytrid infections (Ibelings et al. 2011; Rasconi et al. 2012; Sime‐Ngando 2012). However, it is primarily the presence or absence of suitable hosts that dictates the success of a specific parasite (Ibelings et al. 2004; Sime‐Ngando 2012). In the three water bodies examined in this study, it is evident that this condition had the greatest impact on the outcome of chytrid infections. Each of the water bodies presents itself with a distinct phytoplankton community composition and different biomass. The biomass in Tyniec was 10 times higher compared to Podkamycze 1, twice as high as in Podkamycze 2, and dominated by green algae with *Desmodesmus* spp. being presumably the sole host of chytrid parasites. On the contrary, in Podkamycze 2 which was dominated by cyanobacteria, at the two time points when infection occurred the host species was also *Desmodesmus* spp. and once *Mougeotia* sp. which highlights the fact that in some cases, the most abundant phytoplankton species won't be the most infected one (Gsell et al. 2022). However, infections also occurred on *Aphanizomenon* spp. (including *A. gracile* ) which belongs to the dominant group in Podkamycze 2. Additionally, another crucial trait, namely host specificity (Poulin et al. 2011), could play a significant role in the host–parasite interactions presented in this study. Host specificity has multiple dimensions, including structural, spatial, and phylogenetic factors. For instance, the susceptibility of a host population to parasite infection can vary depending on the compatibility between specific parasite strains and the host genotype (Gsell et al. 2013). Thus, the putative reason for infections that only occur in *Desmodesmus* spp. may have its source in the specificity of the present parasite. Interestingly, chytrids were observed to infect only filamentous cyanobacteria, such as *Aphanizomenon* spp., with no infections occurring in colonial cyanobacteria (e.g., *Microcystis* spp.). In contrast, green algae were susceptible to parasitism, with chytrids infecting both colonial and filamentous forms. It is also worth noting that our observations include chytrids parasitising akinetes, which aligns with findings reported by Gerphagnon et al. (2013).

Cyanobacteria are known hosts to chytrid parasites in both natural and experimental conditions (Rasconi et al. 2012; Wierenga et al. 2022; Xu et al. 2025). These chytrids play a crucial role in aquatic ecosystems by mediating trophic transfer from inedible phytoplankton (e.g., cyanobacteria) to zooplankton through the production of zoospores via the mycoloop (Kagami et al. 2014). These zoospores are rich in polyunsaturated fatty acids (PUFAs) and contain high concentrations of phosphorus‐rich nucleic acids (Kagami, von Elert, et al. 2007), hence facilitating nutrient cycling. Additionally, chytrids can support the aquatic food web dynamics by reducing the complexity and length of filamentous cyanobacterial colonies, making smaller cells more available for grazing (Gerphagnon et al. 2013; Rasconi et al. 2014; Frenken et al. 2018, 2020). However, it may happen that even if the cyanobacteria dominate the biomass, they are not readily infected by chytrids. Instead, chytrids primarily parasitise other phytoplankton groups, such as diatoms and green algae, as well as zooplankton and other fungi (Gleason et al. 2008, 2014). A study by Van den Wyngaert et al. (2022) in Lake Stechlin, Germany, found that fungal infections targeted 10.7% of diatoms, 1.5% of green algae, and only 1% of cyanobacteria. In our study, we did not observe infected cyanobacteria in Tyniec oxbow lake, despite their high biomass. However, IPC on *Desmodesmus* spp. (green algae) was positively correlated with the presence of a cyanobacterial bloom. Field observations showed that during healthy bloom conditions, with active growth, high photosynthesis and a favourable environment, chytrids infect a wider range of phytoplankton, while cyanobacteria infections stay relatively low (Van den Wyngaert et al. 2022). Similar observations were made in Lake Müggelsee, Germany, where commonly occurring cyanobacteria were rarely infected, while diatoms exhibited high infection rates (Gsell et al. 2022). The lack of cyanobacterial infection may be explained by allelopathy, as cyanobacteria are known to produce a variety of secondary metabolites (Agha and Quesada 2014; Nandagopal et al. 2021), which may serve as a defence mechanism against parasitism (Sønstebø and Rohrlack 2011; Rohrlack et al. 2013). This suggests that fungal parasitism of other phytoplankton species may indirectly support the dominance of cyanobacteria by suppressing potential competitors and facilitating their population growth (Marino et al. 2022) or/cyanobacteria could outcompete other species, creating conditions that allow other species to persist and reach the necessary cell densities for chytrid parasitism while avoiding infection themselves.

In our work, the chytrids observed are presumed to be obligate biotrophic parasites, which rely on live hosts for survival and reproduction. This narrowed their ecological niches more than those occupied by their hosts. As such, chytrid occurrence is generally expected to be shaped indirectly through environmental effects on host populations (Kagami, de Bruin, et al. 2007; Marcogliese 2008; Van den Wyngaert et al. 2014). While this ecological dependence means their presence is tightly linked to host availability, our findings suggest that their responses to environmental variables may not always parallel those of the host. The results of our analyses revealed that host biomass was not significantly related to any of the tested parameters, whereas chytrid infection prevalence was associated with cyanobacterial bloom presence, precipitation and temperature. This indicates that chytrids may be responding directly to certain environmental conditions, rather than solely through host‐mediated effects. A well‐documented example of such decoupling is the existence of thermal refuges, where hosts tolerate temperatures at which chytrids cannot establish infections effectively (Gsell et al. 2013, 2023; Rohrlack et al. 2015). Similarly, Gsell et al. (2022) found that long‐term variation in chytrid infection prevalence was significantly associated with temperature and phosphorus, even when infected host biomass remained relatively constant. A similar perspective is offered by Frenken et al. (2017), who proposed that nutrient availability can influence host and parasite performance differently, due to their distinct stoichiometric requirements and physiological demands. These findings support the idea that environmental drivers can shape infection dynamics not only through their impact on host populations, but also via more direct effects on the parasites themselves and highlight the need to consider both host‐mediated and parasite‐specific responses when assessing environmental influences on disease dynamics in aquatic systems.

Podkamycze 1 and 2 ponds are clearly less stable ecosystems compared to Tyniec oxbow lake, partially because of their artificial character (i.e., human‐mediated functioning) resulting in pronounced fluctuations of water level and nutrient concentrations. The pattern found here corresponds to that of Valois and Poulin (2015) whose data synthesis reported that the largest source of variance in infection prevalence was among different water bodies, rather than among different parasite types, years or hosts. In this study, chytrids were assessed and analysed as a single ecological group based on microscopic observations of visible infection structures. While this approach allowed us to capture broad patterns in infection occurrence and prevalence, it does not account for the underlying taxonomic and functional diversity within chytrid communities (Van den Wyngaert et al. 2025). Many chytrids are species‐ or strain‐specific parasites with distinct ecological niches and infection strategies, often tightly linked to the identity and condition of their phytoplankton hosts (Poulin et al. 2011; Frenken et al. 2016; Wierenga et al. 2022). This heterogeneity may influence how different chytrid taxa respond to environmental variability, either directly or through host‐mediated pathways and highlights that these dynamics may be more complex than the resolution of our current dataset. Future studies incorporating molecular tools and species‐level identification will be essential to uncover how chytrid diversity shapes infection outcomes under varying environmental conditions.

## 5 Conclusions

Our findings demonstrate that chytrids infect a wide range of phytoplankton hosts, underscoring their ecological ubiquity in freshwater systems. However, these infections were not consistently present, indicating that chytrid occurrence is highly dynamic and potentially sensitive to environmental fluctuations. Interestingly, cyanobacteria were only weakly infected despite their dominance, which may reflect the influence of defensive traits such as the production of secondary metabolites. As climate change drives warmer temperatures and altered precipitation patterns, conditions favouring chytrid infection may become more frequent. Although chytrids in this study are considered parasites and thus, are dependent on their hosts, they exhibit their own physiological responses and may therefore respond differently to environmental change than their host. Together, these observations highlight the importance of considering both host‐mediated and parasite‐specific responses when assessing environmental influences of disease dynamics in aquatic systems and predicting how parasite–host–environment interactions may shift under future climate scenarios.

## Author Contributions

**Martyna Budziak:** writing – original draft, writing – review and editing, validation, data curation, investigation, conceptualization, visualization, software. **Doris Ilicic:** writing – review and editing, validation, investigation, resources, supervision. **Hans‐Peter Grossart:** investigation, validation, writing – review and editing, supervision, resources. **Wojciech Krztoń:** investigation, validation, writing – review and editing, data curation, software. **Edward Walusiak:** investigation, writing – review and editing, validation, data curation. **Janusz Fyda:** writing – review and editing, validation, investigation, resources. **Elżbieta Wilk‐Woźniak:** supervision, writing – review and editing, validation, investigation, data curation, conceptualization, resources.

## Conflicts of Interest

The authors declare no conflicts of interest.

## Supporting information


**Data S1:** Supporting Information.

## Data Availability

The data that supports the findings of this study are available in the Supporting Information of this article.

## References

[emi470224-bib-0001] Abonyi, A. , J. Fornberg , S. Rasconi , R. Ptacnik , M. J. Kainz , and K. D. Lafferty . 2024. “The Chytrid Insurance Hypothesis: Integrating Parasitic Chytrids Into a Biodiversity‐Ecosystem Functioning Framework for Phytoplankton‐Zooplankton Population Dynamics.” Oecologia 204: 279–288. 10.1007/s00442-024-05519-w. 38366067 PMC10907492

[emi470224-bib-0002] Agha, R. , A. Gross , M. Gerphagnon , T. Rohrlack , and J. Wolinska . 2018. “Fitness and Eco‐Physiological Response of a Chytrid Fungal Parasite Infecting Planktonic Cyanobacteria to Thermal and Host Genotype Variation.” Parasitology 145, no. 10: 1279–1286. 10.1017/S0031182018000215. 29478432

[emi470224-bib-0003] Agha, R. , and A. Quesada . 2014. “Oligopeptides as Biomarkers of Cyanobacterial Subpopulations. Toward an Understanding of Their Biological Role.” Toxins 6, no. 6: 1929–1950. 10.3390/toxins6061929. 24960202 PMC4073138

[emi470224-bib-0004] Agha, R. , M. Saebelfeld , C. Manthey , T. Rohrlack , and J. Wolinska . 2016. “Chytrid Parasitism Facilitates Trophic Transfer Between Bloom‐Forming Cyanobacteria and Zooplankton (Daphnia).” Scientific Reports 6, no. 1: 35039. 10.1038/srep35039. 27733762 PMC5062065

[emi470224-bib-0005] Akhtar, N. , M. I. Syakir Ishak , S. A. Bhawani , and K. Umar . 2021. “Various Natural and Anthropogenic Factors Responsible for Water Quality Degradation: A Review.” Water 13, no. 19: 2660. 10.3390/w13192660.

[emi470224-bib-0006] Altizer, S. , R. S. Ostfeld , P. T. J. Johnson , S. Kutz , and C. D. Harvell . 2013. “Climate Change and Infectious Diseases: From Evidence to a Predictive Framework.” Science 341, no. 6145: 514–519. 10.1126/science.1239401. 23908230

[emi470224-bib-0007] Catlett, D. , E. E. Peacock , E. T. Crockford , et al. 2023. “Temperature Dependence of Parasitoid Infection and Abundance of a Diatom Revealed by Automated Imaging and Classification.” Proceedings of the National Academy of Sciences of The United States of America 120, no. 28: e2303356120. 10.1073/pnas.2303356120. 37399413 PMC10334780

[emi470224-bib-0008] Danz, A. , and C. A. Quandt . 2023. “A Review of the Taxonomic Diversity, Host‐Parasite Interactions, and Experimental Research on Chytrids That Parasitise Diatoms.” Frontiers in Microbiology 14: 1281648. 10.3389/fmicb.2023.1281648. 38029223 PMC10643281

[emi470224-bib-0009] Fong, C. R. , C. J. Gaynus , and R. C. Carpenter . 2020. “Extreme Rainfall Events Pulse Substantial Nutrients and Sediments From Terrestrial to Nearshore Coastal Communities: A Case Study From French Polynesia.” Scientific Reports 10, no. 1: 2955. 10.1038/s41598-020-59807-5. 32076043 PMC7031339

[emi470224-bib-0010] Frenken, T. , E. Alacid , S. A. Berger , et al. 2017. “Integrating Chytrid Fungal Parasites Into Plankton Ecology: Research Gaps and Needs.” Environmental Microbiology 19, no. 10: 3802–3822. 10.1111/1462-2920.13827. 28618196

[emi470224-bib-0011] Frenken, T. , R. Paseka , A. L. González , et al. 2021. “Changing Elemental Cycles, Stoichiometric Mismatches, and Consequences for Pathogens of Primary Producers.” Oikos 130, no. 7: 1046–1055. 10.1111/oik.08253.

[emi470224-bib-0012] Frenken, T. , M. Velthuis , L. N. de Senerpont Domis , et al. 2016. “Warming Accelerates Termination of a Phytoplankton Spring Bloom by Fungal Parasites.” Global Change Biology 22, no. 1: 299–309. 10.1111/gcb.13095. 26488235

[emi470224-bib-0013] Frenken, T. , J. Wierenga , E. van Donk , et al. 2018. “Fungal Parasites of a Toxic Inedible Cyanobacterium Provide Food to Zooplankton.” Limnology and Oceanography 63, no. 6: 2384–2393. 10.1002/lno.10945.

[emi470224-bib-0014] Frenken, T. , J. Wolinska , Y. Tao , T. Rohrlack , and R. Agha . 2020. “Infection of Filamentous Phytoplankton by Fungal Parasites Enhances Herbivory in Pelagic Food Webs.” Limnology and Oceanography 65, no. 11: 2618–2626. 10.1002/lno.11474.

[emi470224-bib-0015] Gerphagnon, M. , D. Latour , J. Colombet , and T. Sime‐Ngando . 2013. “Fungal Parasitism: Life Cycle, Dynamics and Impact on Cyanobacterial Blooms.” PLoS One 8, no. 4: e60894. 10.1371/journal.pone.0060894. 23593345 PMC3625230

[emi470224-bib-0016] Gleason, F. H. , M. Kagami , E. Lefevre , and T. Sime‐Ngando . 2008. “The Ecology of Chytrids in Aquatic Ecosystems: Roles in Food Web Dynamics.” Fungal Biology Reviews 22, no. 1: 17–25. 10.1016/j.fbr.2008.02.001.

[emi470224-bib-0017] Gleason, F. H. , O. Lilje , A. V. Marano , et al. 2014. “Ecological Functions of Zoosporic Hyperparasites.” Frontiers in Microbiology 5: 244. 10.3389/fmicb.2014.00244. 24904557 PMC4035849

[emi470224-bib-0018] Gleason, F. H. , A. V. Marano , A. L. Digby , et al. 2011. “Patterns of Utilization of Different Carbon Sources by Chytridiomycota.” Hydrobiologia 659, no. 1: 55–64. 10.1007/s10750-010-0461-y.

[emi470224-bib-0019] Grossart, H.‐P. , S. Van den Wyngaert , M. Kagami , C. Wurzbacher , M. Cunliffe , and K. Rojas‐Jimenez . 2019. “Fungi in Aquatic Ecosystems.” Nature Reviews Microbiology 17, no. 6: 339–354. 10.1038/s41579-019-0175-8. 30872817

[emi470224-bib-0020] Grossart, H.‐P. , C. Wurzbacher , T. Y. James , and M. Kagami . 2016. “Discovery of Dark Matter Fungi in Aquatic Ecosystems Demands a Reappraisal of the Phylogeny and Ecology of Zoosporic Fungi.” Fungal Ecology 19: 28–38. 10.1016/j.funeco.2015.06.004.

[emi470224-bib-0021] Gsell, A. S. , A. Biere , W. de Boer , et al. 2023. “Environmental Refuges From Disease in Host‐Parasite Interactions Under Global Change.” Ecology 104, no. 4: e4001. 10.1002/ecy.4001. 36799146

[emi470224-bib-0022] Gsell, A. S. , L. N. d. S. Domis , E. van Donk , and B. W. Ibelings . 2013. “Temperature Alters Host Genotype‐Specific Susceptibility to Chytrid Infection.” PLoS One 8, no. 8: e71737. 10.1371/journal.pone.0071737. 23990982 PMC3753301

[emi470224-bib-0023] Gsell, A. S. , J. Wolinska , K. Preuß , et al. 2022. “Long‐Term Trends and Seasonal Variation in Host Density, Temperature, and Nutrients Differentially Affect Chytrid Fungi Parasitising Lake Phytoplankton.” Freshwater Biology 67, no. 9: 1532–1542. 10.1111/fwb.13958.

[emi470224-bib-0024] Huisman, J. , G. A. Codd , H. W. Paerl , B. W. Ibelings , J. M. H. Verspagen , and P. M. Visser . 2018. “Cyanobacterial Blooms.” Nature Reviews Microbiology 16, no. 8: 471–483. 10.1038/s41579-018-0040-1. 29946124

[emi470224-bib-0025] Ibelings, B. W. , A. De Bruin , M. Kagami , M. Rijkeboer , M. Brehm , and E. V. Donk . 2004. “Host Parasite Interactions Between Freshwater Phytoplankton and Chytrid Fungi (Chytridiomycota).” Journal of Phycology 40, no. 3: 437–453. 10.1111/j.1529-8817.2004.03117.x.

[emi470224-bib-0026] Ibelings, B. W. , A. S. Gsell , W. M. Mooij , E. Van Donk , S. Van Den Wyngaert , and L. N. De Senerpont Domis . 2011. “Chytrid Infections and Diatom Spring Blooms: Paradoxical Effects of Climate Warming on Fungal Epidemics in Lakes.” Freshwater Biology 56, no. 4: 754–766. 10.1111/j.1365-2427.2010.02565.x.

[emi470224-bib-0027] Ilicic, D. , J. Woodhouse , U. Karsten , K. Schimani , J. Zimmermann , and H.‐P. Grossart . 2024. “Chytrid Fungi Infecting Arctic Microphytobenthic Communities Under Varying Salinity Conditions.” Scientific Reports 14, no. 1: 25821. 10.1038/s41598-024-77202-2. 39468208 PMC11519490

[emi470224-bib-0028] Kagami, M. , A. de Bruin , B. W. Ibelings , and E. Van Donk . 2007. “Parasitic Chytrids: Their Effects on Phytoplankton Communities and Food‐Web Dynamics.” Hydrobiologia 578, no. 1: 113–129. 10.1007/s10750-006-0438-z.

[emi470224-bib-0029] Kagami, M. , T. Miki , and G. Takimoto . 2014. “Mycoloop: Chytrids in Aquatic Food Webs.” Frontiers in Microbiology 5: 166. 10.3389/fmicb.2014.00166. 24795703 PMC4001071

[emi470224-bib-0030] Kagami, M. , E. von Elert , B. W. Ibelings , A. de Bruin , and E. Van Donk . 2007. “The Parasitic Chytrid, Zygorhizidium, Facilitates the Growth of the Cladoceran Zooplankter, Daphnia, in Cultures of the Inedible Alga, Asterionella.” Proceedings of the Royal Society B: Biological Sciences 274, no. 1617: 1561–1566. 10.1098/rspb.2007.0425. PMC2176168 17439852

[emi470224-bib-0031] Klawonn, I. , S. Dunker , M. Kagami , H.‐P. Grossart , and S. Van den Wyngaert . 2023. “Intercomparison of Two Fluorescent Dyes to Visualize Parasitic Fungi (Chytridiomycota) on Phytoplankton.” Microbial Ecology 85, no. 1: 9–23. 10.1007/s00248-021-01893-7. 34854932 PMC9849195

[emi470224-bib-0032] Komárek, J. 2013. “Cyanoprokaryota: 3rd Part: Heterocystous Genera.” In Süßwasserflora Von Mitteleuropa, edited by B. Büdel , G. Gärtner , L. Krienitz , and M. Schagerl , vol. 19/3. Springer Spektrum.

[emi470224-bib-0033] Krztoń, W. , J. Kosiba , A. Pociecha , and E. Wilk‐Woźniak . 2019. “The Effect of Cyanobacterial Blooms on Bio‐ and Functional Diversity of Zooplankton Communities.” Biodiversity and Conservation 28, no. 7: 1815–1835. 10.1007/s10531-019-01758-z.

[emi470224-bib-0034] Krztoń, W. , E. Walusiak , and E. Wilk‐Woźniak . 2025. “Cyanobacterial Bloom Causes Expansion of Isotopic Niche Areas and Overlap in Crustacean Zooplankton.” Scientific Reports 15, no. 1: 29049. 10.1038/s41598-025-15061-1. 40781504 PMC12334624

[emi470224-bib-0035] Lafferty, K. D. , and E. A. Mordecai . 2016. “The Rise and Fall of Infectious Disease in a Warmer World.” F1000Research 5: 2040. 10.12688/f1000research.8766.1. F1000 Faculty Rev‐2040. PMC4995683 27610227

[emi470224-bib-0036] Lee, H. , K. Calvin , D. Dasgupta , et al. 2023. Climate Change 2023: Synthesis Report. Contribution of Working Groups I, II and III to the Sixth Assessment Report of the Intergovernmental Panel on Climate Change, edited by H. Lee and J. Romero , 1st ed. Intergovernmental Panel on Climate Change (IPCC). 10.59327/IPCC/AR6-9789291691647.

[emi470224-bib-0037] Maier, M. A. , and T. D. Peterson . 2017. “Prevalence of Chytrid Parasitism Among Diatom Populations in the Lower Columbia River (2009‐2013).” Freshwater Biology 62, no. 2: 414–428. 10.1111/fwb.12876.

[emi470224-bib-0038] Marcogliese, D. J. 2008. “The Impact of Climate Change on the Parasites and Infectious Diseases of Aquatic Animals.” Reviews in Science and Technology 27, no. 2: 467–484. 10.20506/rst.27.2.1820. 18819673

[emi470224-bib-0039] Marcogliese, D. J. 2025. “Climate Change and Parasitism in Aquatic Ecosystems.” In Aquatic Parasitology: Ecological and Environmental Concepts and Implications of Marine and Freshwater Parasites, edited by N. J. Smit and B. Sures . Springer Nature Switzerland.

[emi470224-bib-0040] Marino, J. A. , V. J. Denef , G. J. Dick , M. B. Duhaime , and T. Y. James . 2022. “Fungal Community Dynamics Associated With Harmful Cyanobacterial Blooms in Two Great Lakes.” Journal of Great Lakes Research 48, no. 4: 1021–1031. 10.1016/j.jglr.2022.05.007.

[emi470224-bib-0041] McKenzie, V. J. , and A. R. Townsend . 2007. “Parasitic and Infectious Disease Responses to Changing Global Nutrient Cycles.” EcoHealth 4, no. 4: 384–396. 10.1007/s10393-007-0131-3.

[emi470224-bib-0042] Meteomodel.pl . 2024. Pogoda i Klimat . Accessed February 7, 2025. https://meteomodel.pl/aktualne‐dane‐pomiarowe/.

[emi470224-bib-0043] Nandagopal, P. , A. N. Steven , L.‐W. Chan , Z. Rahmat , H. Jamaluddin , and N. I. Mohd Noh . 2021. “Bioactive Metabolites Produced by Cyanobacteria for Growth Adaptation and Their Pharmacological Properties.” Biology 10, no. 10: 1061. 10.3390/biology10101061. 34681158 PMC8533319

[emi470224-bib-0044] Paerl, H. W. , N. S. Hall , and E. S. Calandrino . 2011. “Controlling Harmful Cyanobacterial Blooms in a World Experiencing Anthropogenic and Climatic‐Induced Change.” Science of the Total Environment 409, no. 10: 1739–1745. 10.1016/j.scitotenv.2011.02.001. 21345482

[emi470224-bib-0045] Paerl, H. W. , and J. Huisman . 2008. “Blooms Like it Hot.” Science 320, no. 5872: 57–58. 10.1126/science.1155398. 18388279

[emi470224-bib-0046] Poulin, R. , B. R. Krasnov , and D. Mouillot . 2011. “Host Specificity in Phylogenetic and Geographic Space.” Trends in Parasitology 27, no. 8: 355–361. 10.1016/j.pt.2011.05.003. 21680245

[emi470224-bib-0047] R Core Team . 2022. R: A Language and Environment for Statistical Computing [Computer Software]. R Foundation for Statistical Computing. https://www.R‐project.org/.

[emi470224-bib-0048] Rajarajan, A. , S. Cerbin , K. C. Beng , M. T. Monaghan , and J. Wolinska . 2025. “Warming Increases Richness and Shapes Assemblages of Eukaryotic Parasitic Plankton.” Environmental Microbiomes 20, no. 1: 76. 10.1186/s40793-025-00724-3. PMC12181845 40542417

[emi470224-bib-0049] Rasconi, S. , B. Grami , N. Niquil , M. Jobard , and T. Sime‐Ngando . 2014. “Parasitic Chytrids Sustain Zooplankton Growth During Inedible Algal Bloom.” Frontiers in Microbiology 5: 229. 10.3389/fmicb.2014.00229. 24904543 PMC4033230

[emi470224-bib-0050] Rasconi, S. , M. Jobard , L. Jouve , and T. Sime‐Ngando . 2009. “Use of Calcofluor White for Detection, Identification, and Quantification of Phytoplanktonic Fungal Parasites.” Applied and Environmental Microbiology 75, no. 8: 2545–2553. 10.1128/AEM.02211-08. 19233954 PMC2675195

[emi470224-bib-0051] Rasconi, S. , N. Niquil , and T. Sime‐Ngando . 2012. “Phytoplankton Chytridiomycosis: Community Structure and Infectivity of Fungal Parasites in Aquatic Ecosystems.” Environmental Microbiology 14, no. 8: 2151–2170. 10.1111/j.1462-2920.2011.02690.x. 22309120

[emi470224-bib-0052] Reynolds, C. S. 2004. “Lakes, Limnology and Limnetic Ecology: Towards a New Synthesis.” In The Lakes Handbook: Vol. 1. Limnology and Limnetic Ecology, edited by P. E. O'Sullivan and C. S. Reynolds . Blackwell Science Ltd.

[emi470224-bib-0053] Reynolds, C. S. 2006. The Ecology of Phytoplankton. Cambridge University Press.

[emi470224-bib-0054] Rohrlack, T. , G. Christiansen , and R. Kurmayer . 2013. “Putative Antiparasite Defensive System Involving Ribosomal and Nonribosomal Oligopeptides in Cyanobacteria of the Genus Planktothrix.” Applied and Environmental Microbiology 79, no. 8: 2642–2647. 10.1128/AEM.03499-12. 23396340 PMC3623205

[emi470224-bib-0055] Rohrlack, T. , S. Haande , A. Molversmyr , and M. Kyle . 2015. “Environmental Conditions Determine the Course and Outcome of Phytoplankton Chytridiomycosis.” PLoS One 10, no. 12: e0145559. 10.1371/journal.pone.0145559. 26714010 PMC4703133

[emi470224-bib-0056] Rott, E. 1981. “Some Results From Phytoplankton Counting Intercalibrations.” Schweizerische Zeitschrift für Hydrologie 43, no. 1: 34–62. 10.1007/BF02502471.

[emi470224-bib-0057] Seto, K. , D. R. Simmons , C. A. Quandt , et al. 2023. “A Combined Microscopy and Single‐Cell Sequencing Approach Reveals the Ecology, Morphology, and Phylogeny of Uncultured Lineages of Zoosporic Fungi.” MBio 14, no. 4: e01313‐23. 10.1128/mbio.01313-23. 37486265 PMC10470594

[emi470224-bib-0058] Shearer, C. A. , E. Descals , B. Kohlmeyer , et al. 2007. “Fungal Biodiversity in Aquatic Habitats.” Biodiversity and Conservation 16, no. 1: 49–67. 10.1007/s10531-006-9120-z.

[emi470224-bib-0059] Sime‐Ngando, T. 2012. “Phytoplankton Chytridiomycosis: Fungal Parasites of Phytoplankton and Their Imprints on the Food Web Dynamics.” Frontiers in Microbiology 3: 361. 10.3389/fmicb.2012.00361. 23091469 PMC3469839

[emi470224-bib-0060] Sønstebø, J. H. , and T. Rohrlack . 2011. “Possible Implications of Chytrid Parasitism for Population Subdivision in Freshwater Cyanobacteria of the Genus *Planktothrix* .” Applied and Environmental Microbiology 77, no. 4: 1344–1351. 10.1128/AEM.02153-10. 21169434 PMC3067206

[emi470224-bib-0061] Thongthaisong, P. , M. Kasada , H.‐P. Grossart , and S. Wollrab . 2022. “Critical Role of Parasite‐Mediated Energy Pathway on Community Response to Nutrient Enrichment.” Ecology and Evolution 12, no. 12: e9622. 10.1002/ece3.9622. 36523515 PMC9748242

[emi470224-bib-0062] Thongthaisong, P. , M. Kasada , H.‐P. Grossart , and S. Wollrab . 2025. “Longer Durability of Host‐Parasite Interaction Increases Host Density.” Oikos 2025: e11029. 10.1111/oik.11029.

[emi470224-bib-0063] Utermöhl, H. 1958. “Zur Vervollkommnung Der Quantitativen Phytoplankton‐Methodik.” Mitteilung Internationale Vereinigung Fuer Theoretische Unde Amgewandte Limnologie 9, no. 1: 1–38. 10.1080/05384680.1958.11904091.

[emi470224-bib-0064] Valois, A. E. , and R. Poulin . 2015. “Global Drivers of Parasitism in Freshwater Plankton Communities.” Limnology and Oceanography 60, no. 5: 1707–1718. 10.1002/lno.10127.

[emi470224-bib-0065] Van den Wyngaert, S. , S. Cerbin , L. Garzoli , et al. 2025. “ParAquaSeq, a Database of Ecologically Annotated rRNA Sequences Covering Zoosporic Parasites Infecting Aquatic Primary Producers in Natural and Industrial Systems.” Molecular Ecology Resources 25, no. 6: e14099. 10.1111/1755-0998.14099. 40087979 PMC12225711

[emi470224-bib-0066] Van den Wyngaert, S. , L. Ganzert , K. Seto , et al. 2022. “Seasonality of Parasitic and Saprotrophic Zoosporic Fungi: Linking Sequence Data to Ecological Traits.” ISME Journal 16, no. 9: 2242–2254. 10.1038/s41396-022-01267-y. 35764676 PMC9381765

[emi470224-bib-0067] Van den Wyngaert, S. , O. Vanholsbeeck , P. Spaak , and B. W. Ibelings . 2014. “Parasite Fitness Traits Under Environmental Variation: Disentangling the Roles of a Chytrid's Immediate Host and External Environment.” Microbial Ecology 68, no. 3: 645–656. 10.1007/s00248-014-0434-1. 24863129

[emi470224-bib-0068] Van Wichelen, J. , I. Van Gremberghe , P. Vanormelingen , et al. 2010. “Strong Effects of Amoebae Grazing on the Biomass and Genetic Structure of a Microcystis Bloom (Cyanobacteria).” Environmental Microbiology 12, no. 10: 2797–2813. 10.1111/j.1462-2920.2010.02249.x. 20545742

[emi470224-bib-0069] Wierenga, J. , M. K. Thomas , R. Ranjan , and B. W. Ibelings . 2022. “Complex Effects of Chytrid Parasites on the Growth of the Cyanobacterium *Planktothrix Rubescens* Across Interacting Temperature and Light Gradients.” ISME Communications 2, no. 1: 93. 10.1038/s43705-022-00178-5. 37938757 PMC9723700

[emi470224-bib-0070] Wilk‐Woźniak, E. 2009. “Zmiany Populacyjne w Zbiorowiskach Glonów Planktonowych Oraz Ich Strategie życiowe w Warunkach Ekosystemów Wodnych Sztucznie Zmienionych.” Studia Naturae 55: 1–132.

[emi470224-bib-0071] Wilk‐Woźniak, E. , W. Krztoń , M. Budziak , et al. 2024. “Harmful Blooms Across a Longitudinal Gradient in Central Europe During Heatwave: Cyanobacteria Biomass, Cyanotoxins, and Nutrients.” Ecological Indicators 160: 111929. 10.1016/j.ecolind.2024.111929.

[emi470224-bib-0072] Wolinska, J. , and K. C. King . 2009. “Environment Can Alter Selection in Host‐Parasite Interactions.” Trends in Parasitology 25, no. 5: 236–244. 10.1016/j.pt.2009.02.004. 19356982

[emi470224-bib-0073] Xu, X. , J. Wierenga , M. K. Thomas , and B. W. Ibelings . 2025. “Heterocyst‐Infecting Chytrid Parasites Reduce Nitrogen Fixation and Host Growth Under Nitrogen‐Limiting Conditions in the Cyanobacterium Dolichospermum sp.” Journal of Plankton Research 47, no. 6: fbaf054. 10.1093/plankt/fbaf054. 41070040 PMC12507009

