# Supplementary material for: Phytoplankton Under Pressure: Temperature, Precipitation and Cyanobacterial Blooms as Drivers of Chytrid Infections
Source: Environ Microbiol Rep. 2025 Nov 7;17(6):e70224. doi: 10.1111/1758-2229.70224 (PMC12594634; doi:10.1111/1758-2229.70224)
Supplement: Supplementary file 1 — Data S1: Supporting Information. [file EMI4-17-e70224-s001.docx]

**Supplementary material**

**Phytoplankton under pressure: temperature, precipitation and cyanobacterial blooms as drivers of chytrid infections**

Martyna Budziak*^1^, Doris Ilicic^2^, Hans-Peter Grossart^2,3^, Wojciech Krztoń^1^, Edward Walusiak^1^, Janusz Fyda^4^, Elżbieta Wilk-Woźniak^1^

^1^ Institute of Nature Conservation, Polish Academy of Sciences, al. Adama Mickiewicza 33, 31-120 Kraków, Poland

^2^ Department of Plankton and Microbial Ecology, Leibniz Institute of Freshwater Ecology and Inland Fisheries, Neuglobsow, Germany

^3^ Institute of Biochemistry and Biology, University of Potsdam, Potsdam, Germany

^4^ Institute of Environmental Sciences, Jagiellonian University in Kraków, Kraków, Poland

*Corresponding author: Martyna Budziak, budziak@iop.krakow.pl


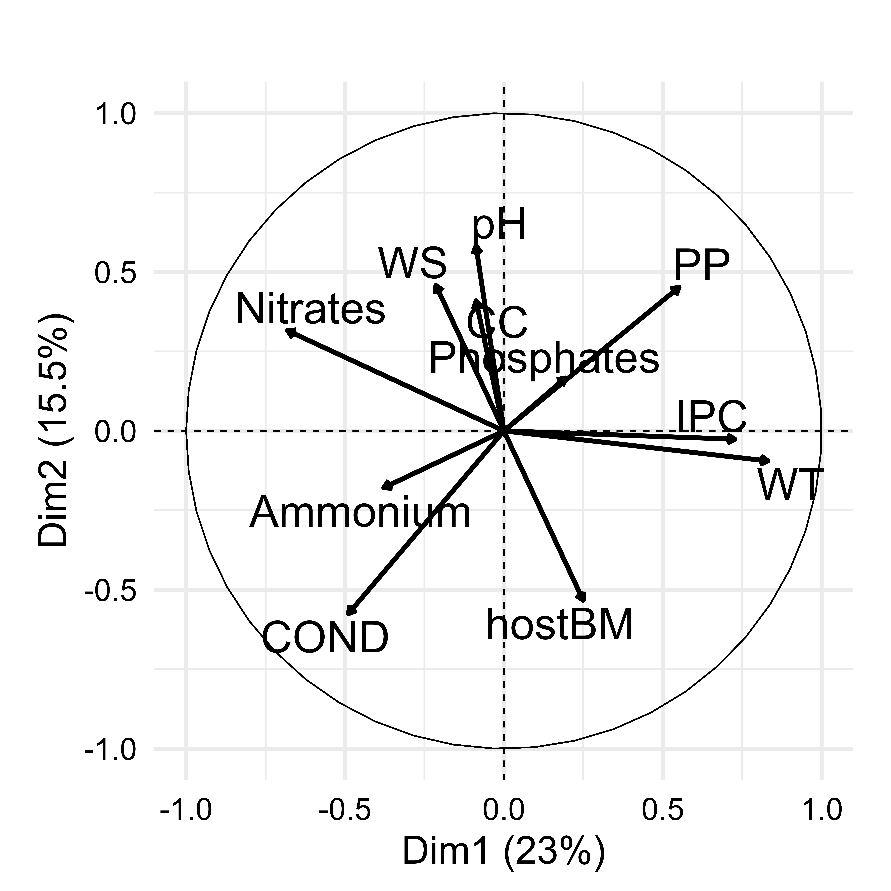


**Figure S1**. PCA for environmental factors in Tyniec oxbow lake. The cumulative explained variation (for axis 1 and 2) is 38.5%. PP = precipitation, WT = water temperature, WS = wind speed, CC = cloud cover, IPC = infection relevance, hostBM = biomass of *Desmodesmus* spp*.*, Ammonium = ammonium ions, Nitrates = nitrate ions, Phosphates = phosphate ions, COND = conductivity.

**Table S1**. Chosen parameters of water present in studied waterbodies.

|  |  | Tyniec | | | Podkamycze 1 | | | Podkamycze 2 | | |
| --- | --- | --- | --- | --- | --- | --- | --- | --- | --- | --- |
|  |  | AV | SD | Min - Max | AV | SD | Min - Max | AV | SD | Min - Max |
| Water temperature [°C] | | | | |  | | |  | | |
|  | 2019 | 18.7 | 5.39 | 11.2 - 25.5 | 17.0 | 4.59 | 10.9 - 23.7 | 18.6 | 5.24 | 11 - 25.6 |
|  | 2020 | 18.6 | 4.63 | 10.5 - 25.1 | 18.5 | 4.35 | 10.2 - 24.7 | 19.1 | 4.25 | 11 - 24.8 |
|  | 2021 | 18.8 | 4.75 | 11.2 - 25 | 18.7 | 5.18 | 10.1 - 23.9 | 18.9 | 4.75 | 10.4 - 24.2 |
|  | 2022 | 17.6 | 4.89 | 9.11 - 22.8 | 17.0 | 4.96 | 8.56 - 22.6 | 17.4 | 4.94 | 8.72 - 22.6 |
|  | 2023 | 18.9 | 4.86 | 9.5 - 24.4 | 18.0 | 4.89 | 8.7 - 24.2 | 18.5 | 4.75 | 8.9 - 22.9 |
|  | 2024 | 19.6 | 4.66 | 10.2 - 25 | 17.9 | 4.57 | 9.06 - 23.7 | 19.4 | 4.88 | 10.3 - 25.4 |
| Conductivity [µS/cm] | | | | |  | | |  | | |
|  | 2019 | 1643 | 135 | 1474 - 1875 | 507 | 46.5 | 436 - 598 | 394 | 43.1 | 326 - 460 |
|  | 2020 | 1851 | 49.3 | 1779 - 1946 | 498 | 61.8 | 370 - 609 | 442 | 50.3 | 377 - 512 |
|  | 2021 | 1542 | 211 | 1275 - 1823 | 490 | 105 | 279 - 562 | 411 | 93.4 | 279 - 523 |
|  | 2022 | 1694 | 137 | 1580 - 1964 | 521 | 48.5 | 452 - 571 | 468 | 63.6 | 365 - 520 |
|  | 2023 | 1638 | 42.5 | 1578 - 1727 | 543 | 40.4 | 466 - 607 | 429 | 61.1 | 342 - 545 |
|  | 2024 | 1411 | 54.9 | 1342 - 1536 | 511 | 59.6 | 364 - 607 | 380 | 62.1 | 307 - 519 |
| pH | | | | |  | | |  | | |
|  | 2019 | 7.74 | 0.405 | 7.23 - 8.43 | 8.25 | 0.323 | 7.42 - 8.78 | 8.27 | 0.269 | 7.84 - 8.63 |
|  | 2020 | 7.6 | 0.257 | 7.24 - 8.19 | 8.17 | 0.285 | 7.66 - 8.48 | 8.13 | 0.240 | 7.7 - 8.52 |
|  | 2021 | 8.31 | 0.351 | 7.84 - 8.7 | 8.64 | 0.416 | 8.1 - 9.2 | 8.73 | 0.441 | 8.22 - 9.3 |
|  | 2022 | 7.64 | 0.349 | 7.4 - 8.38 | 8.19 | 0.12 | 8.05 - 8.41 | 8.42 | 0.133 | 8.08 - 8.45 |
|  | 2023 | 7.94 | 0.273 | 7.55 - 8.34 | 8.06 | 0.216 | 7.65 - 8.32 | 8.24 | 0.280 | 7.76 - 8.64 |
|  | 2024 | 7.9 | 0.223 | 7.56 - 8.3 | 8.07 | 0.115 | 7.84 - 8.26 | 8.14 | 0.264 | 7.72 - 8.7 |
| Nitrate ions [mg/L] | | | | |  | | |  | | |
|  | 2019 | 2.24 | 2.18 | 0.022 - 6.9 | 11 | 1.60 | 9.13 - 15.0 | 2.91 | 2.60 | 0.005 - 8.2 |
|  | 2020 | 1.61 | 1.93 | 0 - 6.67 | 6.82 | 3.18 | 2.94 - 13.6 | 1.90 | 1.69 | 0.008 - 4.34 |
|  | 2021 | 4.71 | 5.20 | 0.059 - 14.5 | 8 | 4.1 | 0.005 - 12.9 | 3.86 | 4.07 | 0.033 - 8.93 |
|  | 2022 | 3.12 | 3.05 | 0.009 - 8.83 | 7.54 | 4.22 | 0.021 - 11.7 | 4.41 | 3.87 | 0.037 - 9.72 |
|  | 2023 | 3.40 | 3.65 | 0.283 - 12.1 | 9.78 | 3.25 | 2.83 - 13.4 | 3.68 | 3.13 | 0.271 - 10.2 |
|  | 2024 | 2.27 | 1.96 | 0.076 - 6.67 | 11.8 | 4.20 | 3.6 - 22 | 2.62 | 2.49 | 0 - 8.47 |
| Ammonium ions [mg/L] | | | | |  | | |  | | |
|  | 2019 | 0.244 | 0.316 | 0.001 - 0.993 | 0.033 | 0.027 | 0.003 - 0.09 | 0.033 | 0.035 | 0.0002 - 0.126 |
|  | 2020 | 0.828 | 0.801 | 0.034 - 2.88 | 0.39 | 0.42 | 0.049 - 1.51 | 0.268 | 0.266 | 0.025 - 0.921 |
|  | 2021 | 0.471 | 0.482 | 0.038 - 1.5 | 0.122 | 0.087 | 0.014 - 0.296 | 0.092 | 0.084 | 0.007 - 0.253 |
|  | 2022 | 0.986 | 0.5 | 0.469 - 1.96 | 0.165 | 0.114 | 0.041 - 0.324 | 0.149 | 0.112 | 0.021 - 0.376 |
|  | 2023 | 1 | 1.05 | 0.111 - 3.71 | 0.352 | 0.748 | 0.041 - 2.69 | 0.109 | 0.085 | 0.002 - 0.318 |
|  | 2024 | 0.578 | 0.786 | 0.024 - 2.7 | 0.148 | 0.159 | 0.013 - 0.535 | 0.025 | 0.027 | 0.002 - 0.083 |
| Phosphate ions [mg/L] | | | | |  | | |  | | |
|  | 2019 | 0.047 | 0.029 | 0.002 - 0.108 | 0.041 | 0.051 | 0 - 0.18 | 0.011 | 0.015 | 0 - 0.037 |
|  | 2020 | 0.179 | 0.505 | 0.001 - 1.85 | 0.13 | 0.143 | 0 - 0.415 | 0.013 | 0.021 | 0 - 0.061 |
|  | 2021 | 0.002 | 0.006 | 0.002 - 0.003 | 0.001 | 0.001 | 0 - 0.002 | 0.005 | 0.008 | 0 - 0.023 |
|  | 2022 | 0.023 | 0.027 | 0.007 - 0.085 | 0.022 | 0.033 | 0 - 0.089 | 0.005 | 0.007 | 0 - 0.155 |
|  | 2023 | 0.38 | 0.119 | 0.284 - 0.716 | 0.266 | 0.111 | 0.064 - 0.398 | 0.109 | 0.057 | 0 - 0.204 |
|  | 2024 | 0.442 | 0.229 | 0.086 - 1.08 | 0.241 | 0.186 | 0.117 - 0.832 | 0.981 | 0.047 | 0 - 0.168 |

Abbreviations: Min-max = range of parameters, AV = average and SD = standard deviation
